# Mapping the Future: Revealing Habitat Preferences and Patterns of the Endangered Chilean Dolphin in Seno Skyring, Patagonia

**DOI:** 10.3390/biology13070514

**Published:** 2024-07-10

**Authors:** Liliana Perez, Yenny Cuellar, Jorge Gibbons, Elias Pinilla Matamala, Simon Demers, Juan Capella

**Affiliations:** 1 Laboratoire de Géosimulation Environnementale (LEDGE), Département de Géographie, Université de Montréal, 1375 Avenue Thérèse-Lavoie-Roux, Montréal, QC H2V 0B3, Canada; yenny.cuellar@umontreal.ca (Y.C.);; 2 Instituto de la Patagonia, Universidad de Magallanes, Av. Pdte. Manuel Bulnes 01890, Punta Arenas 6210427, Chile; jorge.gibbons@umag.cl; 3 Instituto de Fomento Pesquero (IFOP), CTPA Putemún, Castro 5700000, Chile; elias.pinilla@ifop.cl; 4 Whalesound Ltd., C. Lautaro Navarro 1163, 2do piso, Punta Arenas 6201130, Chile; 5 Fundación Yubarta, Calle 34 norte 2E-55 (E107), Cali 760050, Colombia

**Keywords:** Chilean dolphin, habitat modeling, random forest, Seno Skyring Sea, spatial distribution, species distribution models

## Abstract

**Simple Summary:**

Models of species distribution allow us to learn how different environmental factors determine where species are found. Our study focused on the Chilean dolphin in Seno Skyring, Chilean Patagonia. We used three different methods to predict where these dolphins are likely to be found, based on environmental data like water temperature, salinity, and proximity to the coast, as well as human activities like fish farming. We found that dolphins are most commonly found within six kilometers of the coast, in areas with many fish farms. This suggests that the dolphins may be attracted to areas near fish farms. Understanding these patterns is crucial for developing strategies to protect the endangered Chilean dolphin, especially given the large fish-farming industry in Chile. Our research highlights the need for more knowledge and comprehensive conservation efforts to ensure the dolphins’ long-term survival.

**Abstract:**

Species distribution modeling helps understand how environmental factors influence species distribution, creating profiles to predict presence in unexplored areas and assess ecological impacts. This study examined the habitat use and population ecology of the Chilean dolphin in Seno Skyring, Chilean Patagonia. We used three models—random forest (RF), generalized linear model (GLM), and artificial neural network (ANN)—to predict dolphin distribution based on environmental and biotic data like water temperature, salinity, and fish farm density. Our research has determined that the RF model is the most precise tool for predicting the habitat preferences of Chilean dolphins. The results indicate that these dolphins are primarily located within six kilometers of the coast, strongly correlating with areas featuring numerous fish farms, sheltered waters close to the shore with river inputs, and shallow productive zones. This suggests a potential association between dolphin presence and fish-farming activities. These findings can guide targeted conservation measures, such as regulating fish-farming practices and protecting vital coastal areas to improve the survival prospects of the Chilean dolphin. Given the extensive fish-farming industry in Chile, this research highlights the need for greater knowledge and comprehensive conservation efforts to ensure the species’ long-term survival. By understanding and mitigating the impacts of fish farming and other human activities, we can better protect the habitat and well-being of Chilean dolphins.

## 1. Introduction

One of the world’s major producers of fish and fish products, Chile extracts around 1.5 million tons of seafood annually [1], placing the country at the top of the world’s producers [2]. Chile is home to 51 of the world’s 138 marine mammal species, accounting for 36% of the global diversity. [3]. As a result, threats to the conservation of marine mammals are not only ecologically significant in the medium and long term but also pose a potential short-term impact on the Chilean economy. Within the context of international agreements on marine mammal protection to reduce fishing bycatch, there is a particular concern for the conservation status of the Chilean dolphin, *Cephalorhynchus eutropia*, the only endemic cetacean in Chile [4], which is included by Jefferson (2019) among the ten most endangered cetacean species in the world and listed as “Near Threatened” in the Red List IUCN [5] since at the time the total population was estimated to be below 10,000 individuals. However, the absence of data on this important species does not allow for an adequate assessment of the status of the population [4,5].

**Figure 1:**
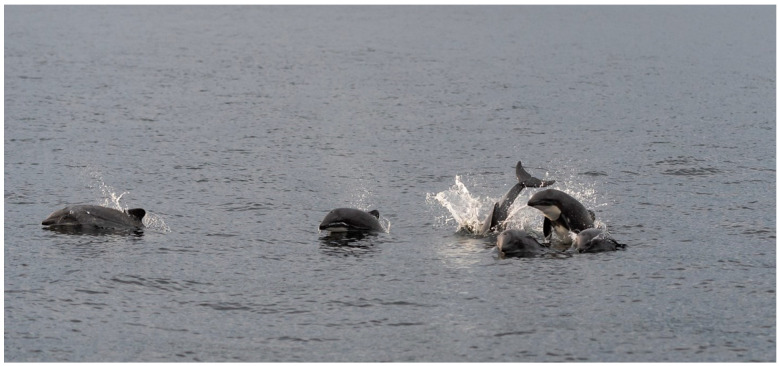
Chilean dolphins off Seno Skyring, Chile (Photo by Simon Demers, December 2019).

The Chilean dolphin, commonly known as the black dolphin, is a small, robust cetacean native to the coastal waters of Chile, typically measuring around 1.7 m in length. These dolphins are characterized by their blunt heads, small dorsal fins, and flippers, with a distinctive coloration of dark gray on the dorsal side and white on the throat and belly, accompanied by notable white patches behind the flippers (Figure 1) [6]. They inhabit cold, shallow coastal waters, including bays, fjords, estuaries, and sometimes rivers, primarily found from Valparaíso in the north to Cape Horn in the south. Their diet consists of fish such as sardines, anchovies, and young salmon, as well as squid and various crustaceans. The distribution of the Chilean dolphin extends from 33° S to the 55°15° S latitude at the southern tip of South America [7,8]. Large-scale genetic and morphometric studies have identified distinct populations, including a northern population along the open coasts, bays, and river mouths of Chile’s midland, Chiloé Island, and the northern Patagonian fjords, as well as populations in the southern fjords and channels region [6,9]. The main threats to the Chilean dolphin include mortality associated with their use as bait for crab fishing [10] and, to a lesser degree, due to human consumption [8], bycatch in boats or coastal gillnet fisheries [11,12], the destruction, contamination, and degradation of their coastal habitat [13,14,15], and boat traffic [15].

**Figure 2:**
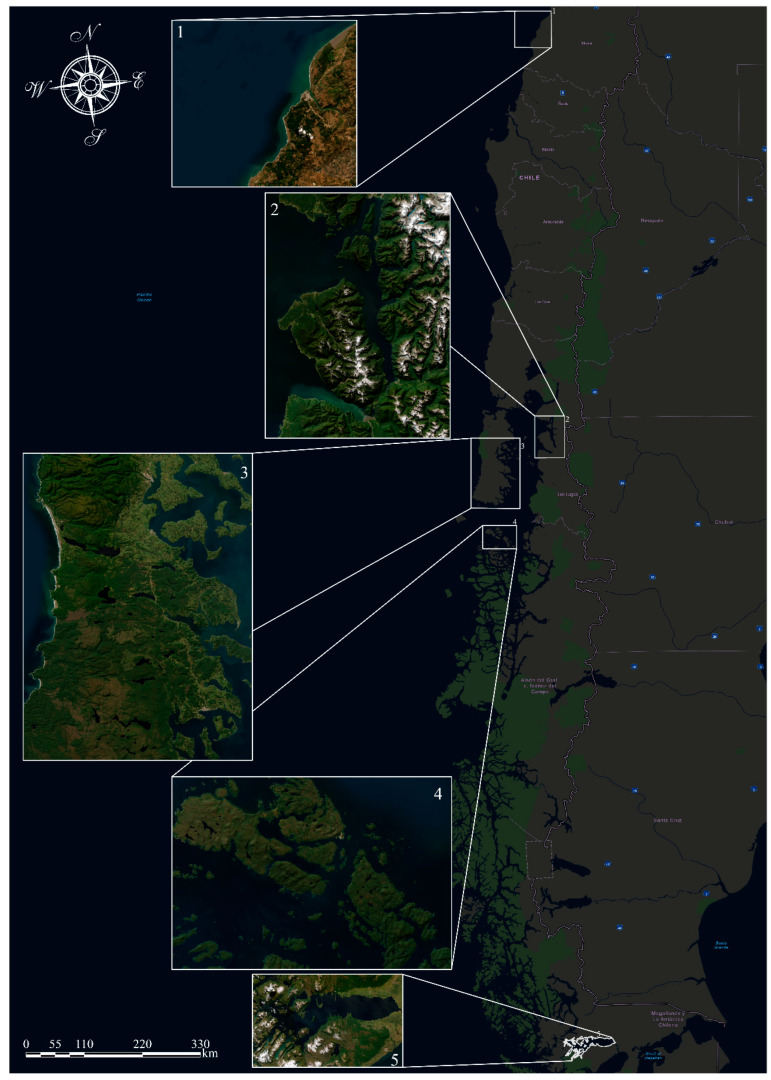
Other studies in Chile about the Chilean dolphin (Cephalorhynchus Eutropia): 1—Maule River, Constitución [6]; 2—Comau and Reñihue Fjords [17]; 3—Chiloé archipelago [15,19]; 4—Guaitecas Islands [21]; 5—Seno Skyring Sea (study area).

Chilean dolphins have been at the core of very few studies (Figure 2) about population abundance, distribution, and ecology. These have focused mainly on the northern population, from the mouth of the Maule River [6], the Valdivia River, and the sheltered bays along Queule [12], to the estuaries, fjords, and channels of localities at the southeast of Chiloé Island [15,16,17,18,19,20], Chiloé north mainland (Ribeiro et al., 2007 [15]), and localities of the north of Aysén and the archipelago of Guaitecas [14,21] and Puyuhuapi channels and fjords [22]. These studies have shown that small resident populations in bays and estuaries characterize the species. On the other hand, habitat-preference modeling studies for Chiloé Island [5,15,19] and the Guaitecas Islands [21] indicate a more likely presence in shallow, fresh, and turbid coastal waters. The concentration of studies in the northern population of Chile only [6] highlights several knowledge gaps regarding the habitat of the southern population of Chilean dolphins and its overall spatial distribution [21].

Limited and outdated information on Chilean dolphins, derived from old studies mainly conducted in the primary channels of the southern Patagonian archipelagos [23], indicate a low frequency and abundance, with the species’ distribution restricted to a few areas. This raises the question, still unanswered after more than two decades, of whether these findings represent the normal distribution and density of the southern population of Chilean dolphins, or if they are the result of a partial and incomplete sampling of this vast region, or if they reflect the impact of mortality associated with their use as bait for crabs [23].

In recent years, the Chilean government’s Undersecretary of Fisheries and Aquaculture has been urgently addressing some critical actions toward the study and conservation of the Chilean dolphin habitat, among them the extrapolation of the north Chilean dolphin population habitat models built for Chiloé and northern Aysén [19,21] to the south of the country, in response to concerns about the state of the dolphin population. While the contribution of the latter study is valued, we recognize several limitations, such as the use of scarce and not-updated information on Chilean dolphins obtained mainly in the primary channels within the Patagonian archipelagos, with very little coverage of the eastern fjords and western coasts [24].

The use of assumptions obtained from northern population habitat models does not consider that the southern Patagonian archipelago offers particularities that are the product of large-scale environmental dynamics. At that latitude, strong westerly winds transport humid air from the Pacific Ocean toward the archipelagic system [25], which translates into a significant latitudinal pluviometric gradient, with precipitation increasing toward the southwest, reaching levels of ~7000 to 8000 mm per year, and decreasing again from the Strait of Magellan toward the south, a longitudinal pluviometric gradient with higher precipitation over the fjords and eastern channels, as the westerly winds ascend as they meet the Andes Mountains, with consequent cooling and precipitation [26,27,28]. Winter snow precipitation over the Andes Mountains at this latitude supports large ice fields, which provide fresh and cold water to the eastern fjords [29]. This also generates a marked gradient in the marine environment, from the colder freshwater of the eastern fjords to the more significant influence of westerly winds and Pacific Ocean waters in the western archipelago.

The study of habitats is essential for understanding a species’ biological requirements and ecological characteristics [30,31,32]. Resources (environmental, biotic, and abiotic) determine the selection of the habitats of a species [33]. Using different species distribution models allows us to assess our knowledge of the ecological factors that define a species’ spatial distribution [34,35,36,37,38]. In this context, advancing our knowledge of different marine mammals underscores the importance of using predictive models. These models first validate hypotheses about population variation and abundance. As our understanding deepens, the resulting descriptive statistics provide the necessary data for these models to accurately predict and map the spatial distribution patterns of species [39]. Ideally, this modeling process should be an integral component of ecological research, incorporating assumptions alongside observational, acoustic, and telemetric data to understand the spatial relationship between a species and its habitat [40].

Given the complexity of the southern Patagonian fjords, this study aims to model the preferred habitat of the Chilean dolphin within a research context where most conservation and research efforts have been focused on northern Chile [5,19,21]. In this context, the recent genetic distinction between Chilean dolphin populations highlights the urgency to expand our understanding of the various ecological features where this endemic species can be observed [9,41] to reduce the knowledge gaps regarding the habitat and overall spatial distribution of the Chilean dolphin [21]. Spatial modeling of preferred habitats is an evolving tool that provides decision makers and scientists with a visual representation of biodiversity-rich areas, allowing us to capture and collect crucial information to understand our ecosystems and the issues of cohabitation caused by the different uses of these spaces [42].

By leveraging systematically updated local data collected over eleven years (2010 to 2021) from the Seno Skyring area in southern Chilean Patagonia, this research seeks to enhance our understanding of the spatial distribution of the Chilean dolphin, *Cephalorhynchus eutropia*, within this specific inland sea, which is part of the Patagonian fjords. Our hypothesis suggests that the distribution of the Chilean dolphin in this region can be comprehended and predicted using a set of ten explanatory variables, including seven environmental factors, two physical factors, and one anthropogenic factor related to human presence. In order to test this hypothesis thoroughly, we employ three of the most prevalent species distribution models (SDMs): random forest (RF), generalized linear model (GLM), and artificial neural network (ANN). Each model is used to generate a predictive raster map of the Chilean dolphin distribution, offering a spatial representation of potential habitats. Identifying the key explanatory variables influencing the distribution of this small, endemic, and endangered cetacean enhances our ecological understanding, crucial for targeted conservation strategies. The use of advanced modeling techniques like RF, GLM, and ANN improves the robustness and reliability of predictions, setting a methodological standard for future marine biology and conservation studies, and providing actionable data for effective protection measures. This is particularly vital given the increasing anthropogenic pressures and environmental changes. The subsequent sections will explore the detailed methodologies used, present the predictive maps generated by the SDMs, and discuss the implications of these findings for the conservation and management of the Chilean dolphin.

## 2. Materials and Methods

### 2.1. Study Area Description

**Figure 3:**
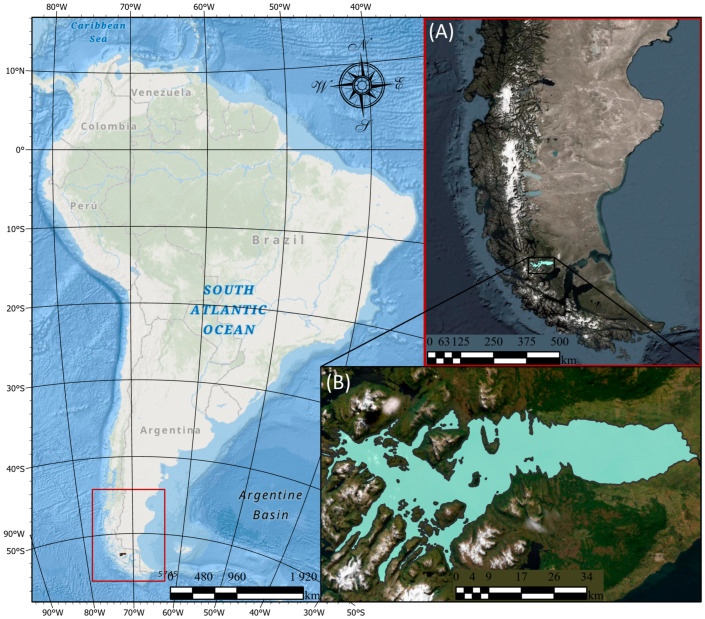
Study area location map: (**A**) Chilean Patagonia and (**B**) Seno Skyring, Chile.

The study area for this research is located in South America, specifically in the southern region of the Chilean southeastern Patagonia fjords. As shown in Figure 3, this area focuses on Seno Skyring, an inland sea, north of Riesco Island in the Magallanes region. North of the Strait of Magellan, two bodies of water form inland seas: Seno Otway and Seno Skyring. The Seno Skyring area covers approximately 1500 km^2^ with a maximum depth of 400 m. The complex geology of Seno Skyring restricts water exchange with the Patagonian fjords and the ocean, resulting in a semi-enclosed estuarine system. This system has minimal connectivity with the ocean and fjords through two very narrow and shallow channels: the Fitzroy Canal to the southeast and the Gajardo Canal to the west.

The marine ecosystem of the fjords connecting the Strait of Magellan is characterized by extreme oceanographic conditions [43]. The region’s unique abiotic and biotic characteristics were partly shaped during the Holocene, specifically by glacial melt. The bathymetric profile, the high precipitation, the marine deposits, and the water supply from the glaciers make Seno Skyring a unique ecosystem. Indeed, the bathymetry and the rivers’ mouth widths make Skyring a practically closed marine system [26]. The high annual precipitation exceeding 5000 mm and the low evaporation rate generate an important freshwater supply with low salinity [29]. Unlike most of Patagonia, where tides largely influence dynamics, Seno Skyring functions differently. In this region, the primary drivers of circulation are the high-energy wind patterns of the Magallanes region to which Seno Skyring, unlike most of the eastern Patagonia fjords, is particularly exposed, as it is located to the west of the highest mountains with icefields and is surrounded (to the north and east) by low hills of the mainland coast and Riesco Island to the south [44].

### 2.2. Methodological Framework

#### 2.2.1. Data

**Figure 4:**
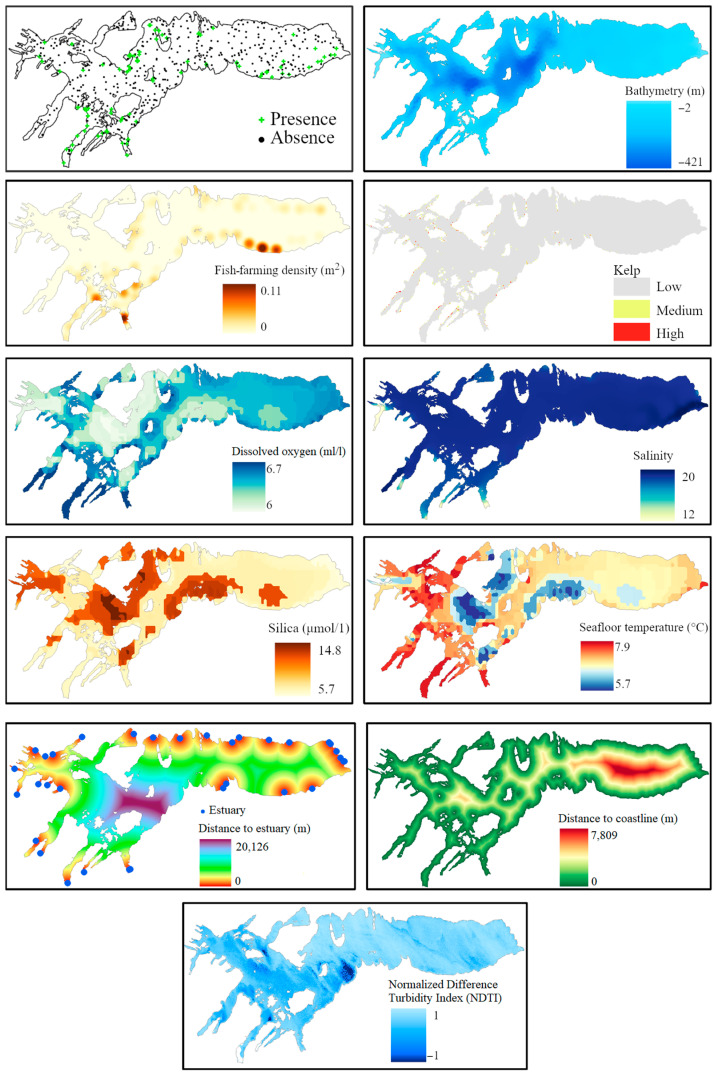
Input data used to obtain the spatial distribution of the Chilean dolphin.

This study uses three different SDMs to predict the likely distribution of the Chilean dolphin in the Skyring area (see Supplementary Materials—Figure S1). The input datasets comprise ten explanatory datasets with a spatial resolution of 100 m × 100 m, and a spatiotemporal layer of Chilean dolphin presence data collected in the field (Figure 4). Presence data were collected using a medium-sized nautical boat as part of several annual marine fauna surveys [22,24] in nine field campaigns between 2010 and 2021; data were tested for spatial autocorrelation in order to account for it in the SDMs used. A total of 350 points of species presence were collected, and 400 points of pseudo-absence entries were generated and included in the model (70% of the data is used for training, while 30% is used to test the model’s classification accuracy); hence, the presence/absence ratio in the final dataset is 7:8. The CHONOS’ oceanographic information system [45] provided explanatory data variables, such as temperature and salinity, derived from a hydrodynamic model detailed in Pinilla et al. (2022) [44]. Specifically, averages from 2016 and 2017 were utilized, due to the lack of more recent datasets. A digital elevation model (DEM) was created using the natural neighbor interpolation method [46] based on nautical charts from the Chilean Navy (SHOA). The selected variables include bathymetry, seafloor temperature, dissolved oxygen, salinity, silica, kelp, fish farms, river mouths, distance to shoreline, and the normalized difference turbidity index (NDTI). Bathymetry maps the underwater topography, crucial for understanding habitat structures, while seafloor temperature influences metabolic rates and prey availability. Dissolved oxygen levels are essential for marine life, affecting ecosystem diversity and abundance. Salinity impacts water density, buoyancy, and osmoregulation, crucial for the dolphin’s habitat preferences. Silica levels influence primary productivity, which supports higher trophic levels, including dolphins. Kelp forests provide essential habitats and food resources, indicating rich biodiversity and productive ecosystems. Proximity to fish farms helps assess anthropogenic impacts and potential human–wildlife conflicts. River mouths, with their high nutrient loads and productivity, attract prey species. Distance to shoreline affects habitat types and human activities, influencing dolphin distribution. Lastly, the NDTI measures turbidity, affecting light penetration and primary productivity, essential for foraging dolphins. These variables collectively capture the environmental and anthropogenic factors influencing the Chilean dolphin’s habitat, leading to accurate predictive models.

The explanatory environmental variables were interpolated using Kriging to provide a continuous layer, and the vector data (i.e., the presence of kelp and the density of fish farms) were transformed from vector to raster format, using the density and point and kernel methods [47]. Moreover, the distance to the river mouths and the shoreline was computed using the Distance and Near tools in ArcGIS Pro [48]. The turbidity of water was calculated using a Landsat 8 satellite image (courtesy of the U.S. Geological Survey) from October 2020, with a 1% cloud cover. The image was preprocessed by applying atmospheric and radiometric correction to convert the digital numbers to light reflectance values. The geometric correction was also performed to reproject the image to the SIRGAS-Chile 2016 UTM zone 19S. To calculate the turbidity index, we followed the methodology applied in other studies [49,50,51]. We calculated the normalized difference turbidity index (NDTI) of the study area using green and red bands. Once all the data were in raster format, resampling was performed to overlay all the layers at the same resolution and extent. A spatial resolution of 100 m by 100 m was chosen to consider the diversity of information sources and the limited availability of datasets for the region. Additionally, the presence- and absence-point layers were produced by overlaying all the Chilean dolphin sightings recorded between 2010 and 2021 in the Seno Skyring area with a random-point layer; this was generated using the random-point layer creation tool available in ArcGIS Pro 2.9.0 [52]. The creation of random absence points in non-inventoried areas allows the model to predict the distribution of the Chilean dolphin throughout the entire study area [53].

#### 2.2.2. Methods for Species Distribution Modeling

This study employed three different species distribution modeling (SDM) approaches: one using traditional statistical methods (GLM) and two utilizing modern machine learning techniques (RF and ANN). Each approach was used independently, and their outputs resulted from individual runs of each algorithm, without combining them into an ensemble.

##### Random Forest (RF)

RF is a machine learning model that extends classification and regression trees (CARTs) [54,55]. RF combines learning methods with a decision tree scheme to create several randomly drawn decision trees to predict categorization or regression outputs. The algorithm works as follows: (1) bootstrap the training data to obtain different subsets; (2) a no-pruned CART is drawn from each bootstrap subset where only one variable (predictor) is selected randomly for the split at each node; and (3) associate the results of all the CARTs with obtaining the predicted results [56]. The number of predictors evaluated at each split was calculated to tune the model. A total of six predictors were fixed. Additionally, the variables’ importance was explored through the GINI index, which measures how important a variable is for estimating the value of the target variable, i.e., Chilean dolphin presence, across all the trees that make up the RF.

##### Generalized Linear Model (GLM)

GLMs are mathematical extensions of linear models that allow non-linearity and non-constant variance structures in the data [57,58]. GLM models are one of the most straightforward parametric approaches to studying species distributions and their relationships with biotic and abiotic covariates [59,60]. In GLMs, the predictor variables are combined to obtain a linear predictor which is associated with the expected value of the target variable through a link function [57]:

(1) $$g\left(E\left(Y\right)\right)=LP=\alpha +X^{T}\beta ,$$

where E(Y) denotes the expected value of the response variable, α is a constant called the intercept, X = (X_1_, …, X_p_) is a vector of the predictor variables, and β = {β_1_, …, β_p_} is the vector of regression coefficients (one for each predictor). The distribution of Y in a GLM can be any familiar exponential distribution, i.e., the binomial, and the link function can be any monotonic differentiable function, i.e., logarithm or logit [57].

##### Artificial Neural Network (ANN)

First proposed in 1943 by McCulloch and Pitts [61], an ANN is a complex model system that involves a network of simple processing elements (artificial neurons) that can display complex global behavior (e.g., habitat site selection based on various environmental variables), governed by the connections between the neurons and associated functions [62]. The ANN used in the study was the nnet of the nnet R package [63], a feed-forward ANN in which vertices can be numbered so that all connections go from a vertex to one with a higher number. The vertices are arranged in layers, connecting only to higher layers [64]. The ANN looks for weights that express the relationship between the layers. Most of the calculations happen in the hidden layers. First, each neuron takes an input from the input layer, multiplies it, and adds it to the initial random weights. Then, the ANN uses some defined transfer and activation functions to make a final prediction in an output layer. The ANN was parameterized using five hidden units in a single layer (selected by cross-validation), with a weight decay equal to 0.01, and repeated 100 times.

#### 2.2.3. Model Evaluation Metrics

##### Area under the Curve (AUC) of a Receiver Operating Characteristic (ROC)

The AUC is the area under a ROC, ranging from 0 to 1, where 1 indicates perfect classification, 0.5 indicates no discrimination, and 0 is not perfect classification. The ROC curve is a graph that displays sensitivity on the y-axis and (1—specificity) on the x-axis, plotted across several classification thresholds., sensitivity as the proportion of correctly predicted observations of species presence and specificity as the proportion of correctly predicted observations of species absence [65]. Higher AUC values depict a better goodness-of-fit model.

##### Root Mean Square Error (RMSE)

Another metric calculated to assess the predictive performance of the models was the RMSE between the model predictions and observations:

(2) $$RMSE=\frac{\sum_{i}^{N}{\left(P_{i}-O_{i}\right)}^{2}}{N}$$

where *P_i_* and *O_i_* are the prediction and observation of the distribution in the sampling site i.

##### True Skill Statistic (TSS)

A metric for evaluating SDM performance, TSS provides an unbiased measure of model accuracy, independent of species prevalence [66]. TSS is very valuable in ecological and conservation research, as it facilitates informed decisions on species management and protection; by incorporating both sensitivity (the model’s ability to accurately predict a species’ presence) and specificity (the model’s ability to accurately predict a species’ absence), TSS ensures a thorough and precise assessment of the SDMs’ predictive capabilities [67]. This dual consideration allows for the assessment of the models’ overall effectiveness in predicting species distribution patterns, thereby improving the reliability of their findings and strategies.

We use the aforementioned SDMs (RF, GML, and ANN) to predict the spatial distribution of the Chilean dolphin across the Seno Skyring at a 100 m × 100 m spatial resolution. The models predict the probability of the presence of Chilean dolphins with values ranging from 0 to 1. So, to define the presence/absence value, we calculated a threshold to convert the probability maps into a binary map (0/1), taking as a base the true positive rate and true negative rate statistics calculated in the AUC. Additionally, to not overestimate the quality of the models, we opted for a robust approach in which we trained the models on a subset of the data and evaluated them on the remaining observations. To train a model, we used 70% of the data and 30% to test the model’s ability to classify the observations correctly. To reduce over-fitting issues, we applied a 5-fold cross-validation method. The entire dataset was divided into five groups, where one was chosen for model testing, and the remaining four were used to train the model. This was repeated in an iterative process until all groups were used to test the model. RStudio software version 2022.07.0 [68] was used to calculate the spatial distribution of the Chilean dolphin. The randomForest [69], caret [70], and stats (R Core Team and contributors worldwide, 2023) packages were used to calculate and perform the model predictions. All the code and datasets used in this study can be openly accessed thru GitHub (https://github.com/ledgeumontreal/chilean_dolphin; accessed on 20 May 2024).

## 3. Results

### 3.1. Model Validation

After the application of the 5-fold cross-validation method, the AUC-ROC and RMSE values (Table 1) for the training and validation dataset were calculated.

The above-presented metrics show an overall good performance for all the models. These performance indicators were used to evaluate the SDMs’ performance because they reflect the degree to which the observed presence/absence points overlap the distribution susceptibility.

Since the AUC is a measurement of the discriminatory capacity of the classification models, this measure for the GLM depicts that the model has low power, compared with the other models, to predict the Chilean dolphin distribution. In contrast, the ANN and RF models performed well, but the random forest model outperformed the ANN following the accuracy metrics used. The TSS, on the other hand, provides a balanced measure of model performance that is independent of species prevalence. Among the three models evaluated for predicting the distribution of the Chilean dolphin in the Seno Skyring, the RF model stands out with the highest TSS value, indicating excellent predictive performance and suitability for identifying critical habitats. The ANN model also demonstrates good performance, while the GLM shows moderate predictive capability. Overall, the RF model is the most reliable, the ANN model serves as a good supplementary tool, and the GLM should be used cautiously with additional validation. A supplementary chart (Figure S2) has been provided to show the ROC curves; in it, sensitivity refers to the proportion of the presences that were correctly classified, and specificity refers to the absences that were correctly classified.

### 3.2. Chilean Dolphin Spatial Distribution Map

**Figure 5:**
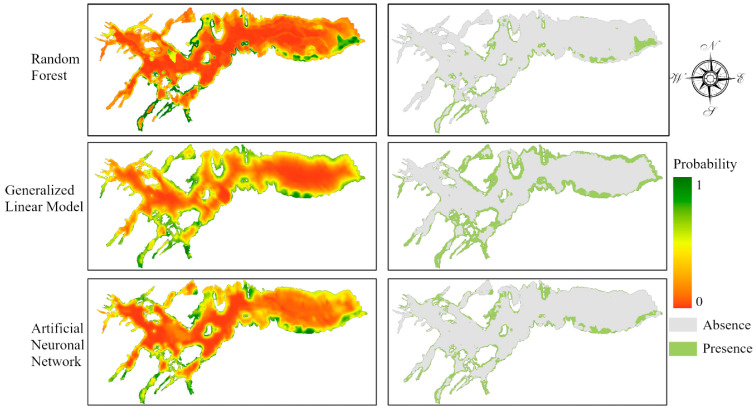
Probability (left) and presence/absence (right) maps of the Chilean dolphin distribution in the Seno Skyring Sea.

Finding the likely spatial distribution of the Chilean dolphin was the primary objective of this study. Figure 5 shows the probability and presence/absence map of the Chilean dolphin’s spatial distribution in the Seno Skyring from the three models applied. The study area distribution patterns predicted by the models were similar, given that the higher probabilities of dolphins’ presence are located along the coastlines, with high turbidity water values, near fish farms. However, the RF model was the one that provided better results, according to the validation results.

From the explanatory variables used with the RF model, it was found that kelp contributes less variance to the distribution of dolphins in the Seno Skyring Sea. In contrast, the distance to the shoreline is the most important variable in the distribution of Chilean dolphins within the RF model. The previous assumption is based on the Gini scores of the RF, i.e., Importance in Table 2, which refers to the contribution each variable had in splitting each node when a tree was built. Hence, it measures the average gain of purity by the splits of each variable in the model. The results show that the distance to the shoreline variable is over 80 times more important than the kelp variable. Furthermore, the density of fish farms is over 36 times more important than the distribution of kelp.

**Figure 6:**
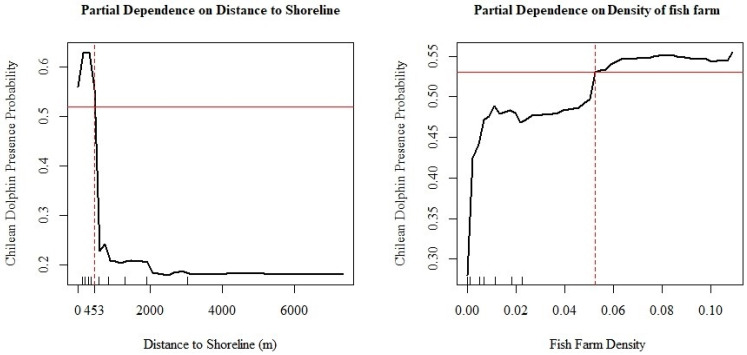
Charts displaying the partial dependence of the most influential predictors. In each chart, the solid red line represents the average partial dependence across all instances, while the dashed line illustrates the Individual Conditional Expectation (ICE) for specific instances. This shows how the predicted outcome varies with changes in the feature for individual instances, helping to visualize the heterogeneity in the model’s predictions.

Based on the importance of variables in the RF model, a graph was created to illustrate the relationship between the distance to the shoreline and the density of fish farms with the distribution of Chilean dolphins. Figure 6 depicts the dependency of these variables. The point at which the red lines intersect indicates, in the case of the distance to the coastline, the furthest point where there is a higher probability of Chilean dolphin presence. The density of fish farms also shows the location from which there is a higher probability of their presence.

## 4. Discussion

This study presents a robust model incorporating ten explanatory variables and several years of data on the Chilean dolphin’s preferred habitat. It identifies ecological factors influencing the spatial distribution of these dolphins in Seno Skyring, an eastern Patagonian fjord located two thousand kilometers south of other studied sites [19,21,71,72]. Our findings demonstrate the effectiveness of SDMs in predicting Chilean dolphin distribution. Although model performance varies, even the lowest-performing model, GLM, achieved an AUC of 0.81. However, the RF model outperformed the others, with a cross-validated AUC exceeding 0.97 and a TSS of 0.91, indicating that the model accurately identifies areas where the species is likely to be found and where it is not. These results align with previous research comparing SDMs for cetaceans [60,73,74].

Dolphin fauna in the eastern Patagonian fjords, particularly Skyring, has been understudied. Earlier research in the larger central channels of the southern Patagonian fjords and the Strait of Magellan reported rare sightings of Chilean dolphins. However, the most extensive, detailed, and continuous (2013–2022) monitoring of marine mammals, which is used in this research, shows contrasting results with those of Gibbons et al. (2002) and Goodall et al. (1997), placing the Chilean dolphin as the main cetacean species, with the highest relative abundances and interannual presence, in three fjords [75,76,77,78,79,80,81,82].

Our results confirm a higher probability of Chilean dolphin occurrence in coastal and shallow waters, consistent with previous models from northern Chiloé Island [5,19] and the Guaitecas Islands [21] in the northern Patagonian fjords of Chile [19,20,21,41,83], and also matches the findings of the few other studies conducted off the open mainland coast of central Chile [9]. However, unlike other studies, our results do not confirm the significance of proximity to river mouths for dolphin distribution [6,15,19,21]. Seno Skyring’s water uniformity, influenced by limited oceanic inflow, high wind energy, and low river flow, sets it apart from other areas in Patagonia.

The RF model implemented here uniquely shows a strong positive relationship between Chilean dolphin occurrence and proximity to salmon farms, a correlation not observed in prior studies [15,19]. However, with the current data, it is unclear whether this relationship indicates a direct attraction or coincidental habitat preference. More information on dolphin behavior is needed to establish a causal explanation. These findings are important as Seno Skyring’s distinct climatic and oceanographic conditions differ significantly from most of the Patagonian canal and fjord system [27]. This progress is vital for creating a matrix prediction map to compare sightings between northern and southern Chilean dolphin populations [6].

In regard to our methodological approach, this research aligns with [36], who emphasized the need of standardized methodologies in cetacean habitat studies, and [39], who highlighted the superior performance of ensemble models, resonating with our RF model’s results. Ref. [40] discussed integrating data across species and scales, and our long-term data collection enhances the understanding of habitat preferences. Ref. [43] examined the influence of marine fronts and physical processes on marine life, aligning with our findings on Seno Skyring’s water homogeneity. Ref. [26] described Seno Skyring’s paleoecological evolution, providing context for our environmental observations. Ref. [41] focused on genetic analysis and population structure, complementing our ecological approach, while [42] used habitat modeling for conservation, similar to our study’s implications for managing fish farm and vessel traffic risks.

While our study provides valuable insights, it also acknowledges limitations. Forecasting the environmental conditions shaping a species’ spatial distribution involves challenges, particularly in the spatial scope of modeling this unique species’ habitat. Extending our findings to similar regions, especially in southern Chile, is beneficial. The complexity of marine ecosystems requires numerous studies and modeling efforts to train the algorithm effectively. Additionally, data granularity is a constraint. Raster data manipulation to fill spatial gaps reduces accuracy. Converting all data to raster format and resampling to a uniform resolution and extent (100 m by 100 m) was necessary due to varied information sources and limited datasets for the region.

**Figure 7:**
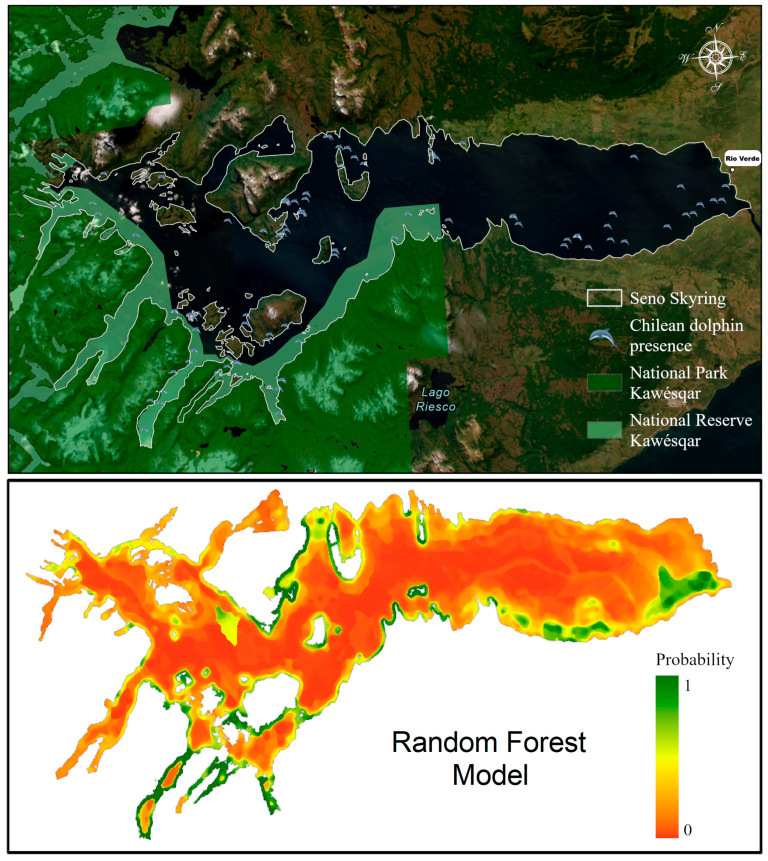
Maps presenting the boundaries of the National Park Kawésqar and the National Reserve Kawésqar in comparison with the results of the RF species distribution model of the Chilean dolphin distribution in the Seno Skyring Sea.

For management purposes, it is crucial to evaluate the risks and impacts, both direct and indirect, of the overlap between fish farms and Chilean dolphin distribution in Seno Skyring. Potential risks include collisions from frequent boat activity, behavioral changes and stress due to disturbances and noise pollution, and possible health effects [84,85]. The preference for coastal areas may reduce vessel collision risks. However, the Fitzroy Channel, the only navigation route into Seno Skyring, presents significant risks due to the presence of Chilean dolphins and Commerson’s dolphins [75,86]. Furthermore, Figure 7 shows the current boundaries of both, the National Park Kawésqar and the National Reserve Kawésqar, and the outputs of the RF species distribution model of the Chilean dolphin; it is important to highlight that there is still a big portion of the study area that has no status of protection, which in the near future could represent a great threat to a decreasing population of an endemic species.

## 5. Conclusions

This research aims to forecast the potential spatial distribution of the Chilean dolphin in Seno Skyring, a fjord located in southern Chilean Patagonia. Utilizing three species distribution models (SDMs)—random forest (RF), generalized linear model (GLM), and artificial neural network (ANN)—we evaluated the ecological factors influencing dolphin distribution. The RF model demonstrated the highest accuracy, with an area under the curve (AUC) exceeding 0.95 and a true skill statistic (TSS) of 0.91. The results indicate that proximity to the shoreline and the density of fish farms are critical factors in determining the distribution of Chilean dolphins in the area.

Our study highlights the key ecological factors that influence the spatial distribution of Chilean dolphins in Seno Skyring, an eastern Patagonian fjord significantly distant from other studied sites. Using ten explanatory variables and multiple years of data, we employed SDMs to predict dolphin distribution, and the RF model achieved the highest performance. These results not only corroborate previous cetacean SDM studies but also align with standardized methodologies in cetacean habitat studies, the superior performance of ensemble models, the integration of multiscale data, and the influence of marine fronts and physical processes on marine life.

Our findings provide critical insights for conservation efforts, particularly in managing risks from fish farms and vessel traffic. The study holds significant implications for the conservation of the Chilean dolphin by enhancing our understanding of their preferred habitats and spatial distribution patterns. The findings provide valuable insights for decision makers and scientists for formulating effective conservation strategies and policies to safeguard the species. Given that Chile is a major global producer of fish and fish products, it is crucial to account for the potential impacts of anthropogenic disturbances, such as maritime traffic, tourism, fishing practices, and aquaculture, on marine mammal populations. This study underscores the urgency of expanding our knowledge of the ecological characteristics of the southern Chilean dolphin population to develop comprehensive conservation measures.

## Figures and Tables

**Table 1:** AUC-ROC values for the training and test dataset applying the three models.

Models	AUC			
	Training	Validation	RMSE	TSS
RF	1	0.97	0.22	0.91
GLM	0.82	0.81	1.70	0.52
ANN	0.94	0.88	0.37	0.68

**Table 2:** Importance of variables in the RF model.

Variable	Importance
**Distance to shoreline**	**111.4**
**Fish farm density**	**50**
Salinity	48
Bathymetry	35
Turbidity	31
River mouths	28
Dissolved oxygen	26
Seafloor temperature	21
Silica	20
Kelp	1.4

## Data Availability

The raw data supporting the conclusions of this article will be made available by the authors on request.

## Supplementary Materials

The following supporting information can be downloaded at https://www.mdpi.com/article/10.3390/biology13070514/s1, Figure S1: Workflow diagram of the methodological framework of this study, Figure S2: ROC curves along the AUC values for each model (AUC values in Table 1).
