# Supplementary material for: Mapping the Future: Revealing Habitat Preferences and Patterns of the Endangered Chilean Dolphin in Seno Skyring, Patagonia
Source: Biology (Basel). 2024 Jul 10;13(7):514. doi: 10.3390/biology13070514 (PMC11274189; doi:10.3390/biology13070514)
Supplement: Supplementary file 1 [file biology-13-00514-s001.zip › biology-3048965-supplementary.pdf]

Supplementary Material

Mapping the Future: Revealing Habitat Preferences and Patterns of the Endangered Chilean Dolphin in Seno Skyring, Patagonia

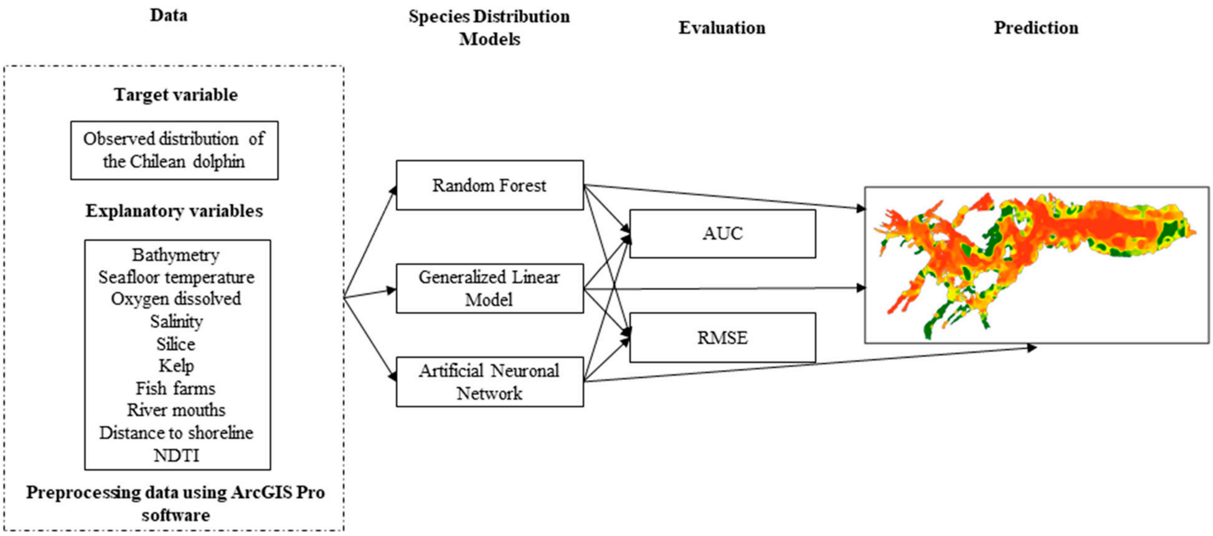

Figure S1. Workflow diagram of the methodological framework of this study

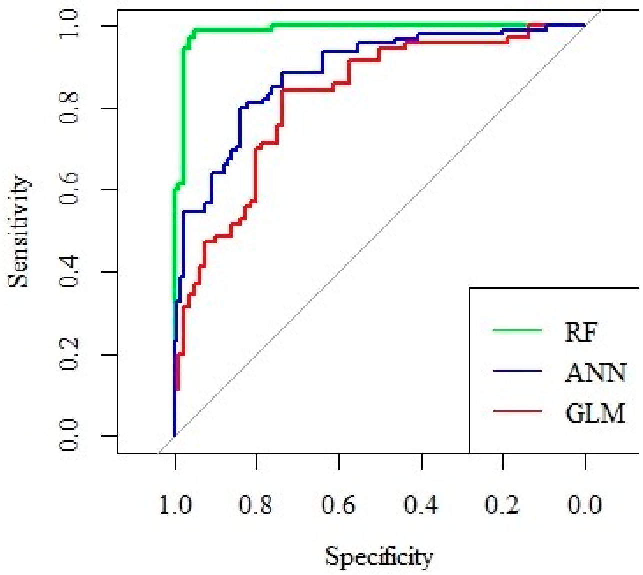

Figure S2. ROC curves along the AUC values for each model (AUC values in Table 1S).
